# Utilization of Maillard reaction in moist-dry-heating system to enhance physicochemical and antioxidative properties of dried whole longan fruit

**DOI:** 10.1016/j.heliyon.2021.e07094

**Published:** 2021-05-22

**Authors:** Chalermkwan Somjai, Thanyaporn Siriwoharn, Kanokwan Kulprachakarn, Supakit Chaipoot, Rewat Phongphisutthinant, Pairote Wiriyacharee

**Affiliations:** a Division of Product Development Technology, Faculty of Agro-Industry, Chiang Mai University, Chiang Mai 50100, Thailand; b Division of Food Science and Technology, Faculty of Agro-Industry, Chiang Mai University, Chiang Mai 50100, Thailand; c School of Health Sciences Research, Research Institute for Health Science, Chiang Mai University, Chiang Mai 50200, Thailand; d Science and Technology Research Institute of Chiang Mai University, Chiang Mai 50200, Thailand; e Center of Excellent in Microbial Diversity and Sustainable Utilization, Faculty of Science, Chiang Mai University, Chiang Mai 50200, Thailand

**Keywords:** Maillard reaction, Non-enzymatic glycosylation, Rare sugar, Sugar-protein conjugates, Antioxidant activity, Polysaccharide, Aging process

## Abstract

This research aimed to enhance the physicochemical and antioxidant properties of dried whole longan fruit using Maillard reaction or non-enzymatic glycosylation (glycation) in a moist-dry-heating system at 60 °C with approximately 75% relative humidity for 5–50 days. During Maillard reaction, the browning index (BI) of the fruits increased significantly while lightless, redness and yellowness decreased. Interestingly, the rare sugars especially D-psicose and D-allose gradually increased by 2–3 folds when compared to the initial Maillard reaction. The development of D-mannose was additionally established through the glycation. The degree of glycation increased with the decrease of free amino acid, suggesting that conjugation of sugar with amino acids was involved. SDS-PAGE confirmed that the high molecular weight (HMW) of conjugated sugar-amino acid was the Maillard reaction product. The antioxidative properties including DPPH and ABTS radical scavenging activities, also ferric reducing antioxidant power (FRAP) were also increased as Maillard reaction progressed, which showed the activities in the range of 43.2–94.1 mg GAE/100 g dry basis, 0.23–3.09 g TE/100 g dry basis, and 0.35–5.95 g FeSO_4_/100 g dry basis, respectively. This study demonstrated a practical approach of Maillard reaction for the development of dried longan fruit with high antioxidative properties.

## 1 Introduction

Longan (*Dimocarpus longan* Lour. cultivar Edor) is an economic fruit of the northern part of Thailand, which frequently has a problem of product oversupply and price slumps. According to a dried whole longan manufacturer and exporter (Honeyqueen Co., Ltd. Lamphun, Thailand), about 20 metric tons of the product from the factory have been a massive surplus for consumer demand since 2017–2020. This was mainly due to unacceptable changes in physical qualities of fruit pulp color during processing and storage. Although these could be delayed in chilled storage, an increase of energy use offset the profits. Otherwise, ancient Chinese people have been using the dark dried longan pulp in traditional medicine recipes for treating some serious health problems (e.g., amnesia, insomnia, and heart palpitation) [1, 2]. Both the process and storage duration can affect the chemical change of bioactive substances presented in longan pulp. Some studies reported that there were three main groups of bioactive compounds in longan pulp which were saccharides, phenols, and liposoluble constituents. Some identified mono/di-polysaccharide components in longan extract include glucose, sucrose, fructose, mannose, arabinose, xylose, galactose, and possibly some rare sugar molecules [3, 4, 5]. Furthermore, the sugar-protein conjugates (SPC) (an active macromolecule) in dried longan pulp extract also presented activities of antioxidant, potential anticancer, and immunomodulatory [2, 4, 6, 7, 8].

The Maillard reaction or non-enzymatic glycosylation is recognized as one of the main reactions in dried longan fruit processing and storage, which could relate to a complex between amino acids and reducing sugars with covalent bond. The Maillard reaction products (MRPs) and/or the SPC of dried fruit products are exhibited during manufacturing, warehousing and shipping of the product [2, 3]. The Maillard conjugates in early stage or the SPC and melanoidin are provided with sensory characteristics, which can be utilized in food products for color, flavor, and taste improvement [9, 10, 11]. Moreover, the SPC could effectively enhance the antioxidant, antitumor, antihypertensive, and immune-stimulating activities through *in vitro* techniques [10, 12, 13]. Several researches in food model systems and products have showed the functional properties enhancement of protein/peptide-saccharide conjugates via Maillard reaction, for instance, solubility, emulsifying ability, thermostability, foaming capacity, gelation, viscosity, and heat-sealing ability [14, 15, 16]. Furthermore, Sun *et al.* [17] reported that protein glycated with rare sugar (D-allose) had a strong antioxidant activity for scavenging free radicals and delaying deterioration due to the oxidation reaction. The SPC obtained under controlled conditions (temperature, heating time, moisture, pH, substrate concentration) could additionally improve in biological and functional properties [12, 16, 18, 19].

Rare sugars can be found in nature in form of monosaccharides. Regardless of their small quantities, they have an excellent potential for practical utilization as bioactive ingredients for drug formulation [20, 21]. Some rare sugars (D-psicose or D-allulose, D-tagatose) can serve as low calorie sweeteners with low glycemic index that are suitable for diabetic patients and obese persons. D-allose is one of the rare sugars which has an inhibitory effect on non-communicable diseases (NCDs), particularly in cancers, and it also used as an anti-inflammatory agent for surgery treatment [20, 22]. Typically, some rare sugars could be produced using two classes of the enzyme (epimerases and isomerases) in which D-fructose and D-glucose can be used as raw substrates. However, a non-enzymatic conversion of these sugars to rare sugar molecules has also been performed by an alkali-catalyzed and a metal-catalyzed isomerization reaction [23, 24]. The enolization of D-fructose anion isomerization under ordinary heating conditions involves D-psicose formation, which is also known as the *Lobry de Bruyn-Alberda van Ekenstein* rearrangement [25, 26].

The objective of this research was to investigate the changes in physicochemical and antioxidant qualities of dried longan pulp prepared by dry-heat treatment of dried whole longan under temperature-controlled with high relative humidity, ranging from 5 to 50 days of aging time. In addition, SDS-PAGE was performed to explain the conjugated compounds occurring after the aging process. Thus, overall results would be useful information to gain a thorough understanding of dried whole longan products relating to the glycosylation/Maillard reaction, which might provide as superfood product with high antioxidant content.

## 2 Materials and methods

### 2.1 Materials and chemicals

Fresh longan fruits cultivar Edor with size code 2 (more than 2.2–2.5 cm in diameter) was purchased from a local market in Lamphun province in 2019. A standard solution of amino acids mixture (type H), which contained 17 common amino acids (e.g., Asp-aspartic acid, Thr-threonine, Ser-serine, Glu-glutamic acid, Pro-proline, Gly-glycine, Ala-alanine, Cys-cysteine, Val-valine, Met-methionine, Ile-isoleucine, Leu-leucine, Tyr-tyrosine, Phe-phenylalanine, His-histidine, Lys-lysine and Arg-arginine) was purchased from Wako Pure Chemical Corporation, Osaka, Japan. Eight types of sugar standards used for analysis were D-psicose, D-glucose, D-fructose, D-xylose, D-rhamnose, D-allose, D-mannose, and sucrose (Sigma-Aldrich Singapore, Singapore). HPLC grade methanol and acetonitrile were purchased from RCI, Labscan, Bangkok, Thailand. All solutions and dilutions were performed using deionized water, which was generated using a Milli-Q water system (Zeneer up 900, Seoul, Korea).

### 2.2 Preparation of black dried longan pulp (BDLP) and BDLP extract

Briefly, whole longan fruits were dried in a hot air dryer using a drying condition of Honeyqueen Co., Ltd. (Lamphun, Thailand), with the final requirements for the pulp: 14–17% moisture content and about 0.5–0.6 water activity (a_w_) according to Thai agricultural standard (TAS 10–2006) [27]. Then, 500 g dried whole longan (control sample) were incubated at 60 °C in desiccators previously equilibrated at ~75% relative humidity (NaCl saturated solution) with aging time of 5, 10, 15, 20, 25, 30, 40, and 50 days. The treated samples were peeled and seeded to obtain only the pulp. Black dried longan pulp (BDLP) samples were packed immediately in a polyethylene bag under vacuum and then stored at -18 °C in the freezer until analysis.

Both the control and treated pulp samples were soaked and blended with deionized water at a solid-liquid ratio of 1:10 using a hand blender (800W, Philips, Thailand) for 2 min. The mixture was sonicated for 10 min with an ultrasonic bath (frequency: 37 kHz, ultrasonic power effective: 80W) (Elmasonic S 30H, Elma Shmidbauer GmbH, Singen, Germany). To reduce Maillard reaction effect during extraction, the temperature was controlled at 25 °C and then centrifuged at 8,000 rpm for 15 min at the same temperature (UNIVERSAL 320R, Hettich, Massachusetts, U.S.). The supernatant was filtrated through a Whatman No. 4 filter paper and lyophilized to get dried longan extract for physicochemical analysis. All extracts were kept at -18 °C to avoid glycation during storage.

### 2.3 Determination on physical qualities of BDLP

The color of the dried longan pulps was determined using a colorimeter (CR-400, Konica Minolta, Japan) in *L∗*, *a∗* and *b∗* values. The measurement of the browning index (BI) followed a modified method of Baloch, Khan, & Baloch [28] and Cernîsev [29], Five g of dried longan pulp was put in a 100 mL beaker with approximately 30 mL of acetic acid (2%, v/v) for 10 min, then all samples were homogenized with a hand blender for 30 s. The mixture solutions were filtered with a filter paper (Whatman No. 4). Appropriate dilutions (10-fold) of the longan filtrates were made using distilled water, and the absorbance was measured at 420 nm against 2% acetic acid (blank) using a UV spectrophotometer (UV1800; Shimadzu, Japan). The analysis was performed in triplicate on each sample and the average values were taken.

### 2.4 Chemical analysis of BDLP extracts

#### 2.4.1 Reducing sugar content

A modified dinitrosalicyclic acid (DNS) method of Gandhi *et al.* [30] was used for DNS reagent preparation. Standard curves were plotted by using the glucose solution (0.2–1.0 mg/mL). One mL of longan pulp extract and 4 mL of DNS reagent were mixed in a test tube and covered with aluminium foil to avoid the loss of liquid due to evaporation. The mixture was heated in boiling water (90 °C) bath for 5 min. Thereafter, 10 mL of distilled water was added to stabilize the color. The test tube was cooled to room temperature in a cold-water bath, then the absorbance of the sample solution was measured at 550 nm against a blank (replacing the longan extract with distilled water) by spectrophotometer.

#### 2.4.2 Sugars contents

**Figure 1:**
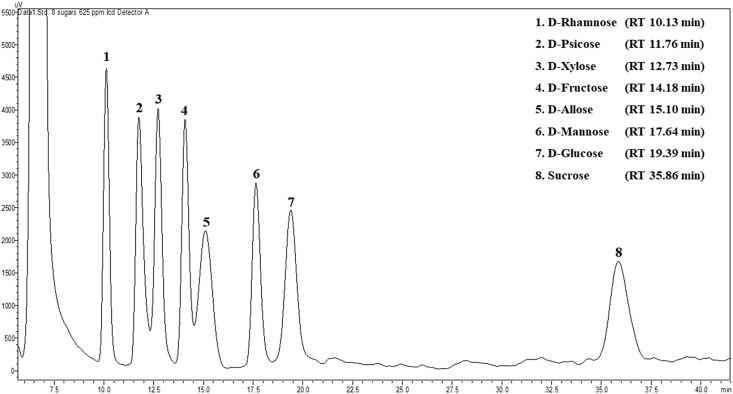
HPLC chromatogram of eight standard sugars (D-rhamnose, D-psicose, D-xylose, D-fructose, D-allose, D-mannose, D-glucose, and sucrose).

According to a method of Shodex™ capture the Essence [31], a standard of eight sugars the sample extracts were analyzed by high-performance liquid chromatography (HPLC) with a Shodex HILICpak VG-50 4E column (4.6 mm I.D. × 250 mm length, Showa Denko, Tokyo, Japan), and a refractometer (Shimadzu, Kyoto, Japan). A mixture of acetonitrile, methanol, and water (85:10:5, v/v) was used as an eluent at a flow rate of 0.6 mL/min. The column temperature was regulated at 50 °C using a CTO-20AC column oven (Shimadzu). The BDLP extract was diluted 20-fold with acetonitrile (50:50, v/v) and then filtered through a 0.20 μm membrane filter. A 10 μL aliquot was injected for each run. The chromatogram of each sugar standards with concentration of 625 ppm was shown in Figure 1. For each determination, three replicates were obtained and an average was taken.

#### 2.4.3 Proteogenic amino acids analysis

Seventeen amino acids were determined by HPLC using a method of Masuda & Yamamoto [32] with post-column reaction. A Shim-pack Amino-Na column (100 mm × 6.0 mm I.D., 5 μm; P/N: 228-18837-91, Shimadzu, Japan) was used as a column to separate the amino acids. The Na type mobile phase solution consisted of three types of solutions A, B, and C. The composition of mobile phases A and B were sodium citrate buffers with pH 3.23 and pH 10.0, respectively. An aqueous solution of 0.2 M sodium hydroxide was used as a mobile phase C. An *o*-phthalaldehyde (OPA) and N-acetylcysteine were prepared as the reaction reagents, resulting in a detection of fluorescent derivatives. The column oven temperature was maintained at 60 °C, the flow rate at 0.4 mL/min, injection volume at 10 μL, and a fluorescence detector was used.

#### 2.4.4 Degree of glycation (DG) by OPA method

The measurement of DG was determined by a slightly modified OPA assay, which was calculated based on the loss of amino groups [33, 34]. The OPA reagent was prepared by mixing 0.2 g of OPA (dissolved in 5 mL of absolute ethanol), 125 mL of 0.1 M sodium tetraborate buffer (pH 9.75), 0.5 mL of β-mercaptoethanol, and 12.5 mL of 10% (w/v) sodium dodecyl sulfate (SDS), and then diluting to a final volume of 250 mL with distilled water. BDLP extract solution (50 μL) was incubated with 3 mL of OPA reagent at 25 °C for 2 min. The absorbance at 340 nm was measured by UV-vis spectrophotometer. A calibration curve was obtained using 0.05–1 mM L-lysine. The DG was calculated according to Eq. (1):

(1) DG (%) = (*A*_*0*_–*A*_*t*_)/*A*_*0*_ × 100

where *A*_*0*_ is an absorbance of the control sample, and *A*_*t*_ is the absorbance after glycation for *t* day.

#### 2.4.5 Sodium dodecyl sulphate-polyacrylamide gel electrophoresis (SDS-PAGE) analysis

Gel electrophoresis was carried out according to Laemmli's procedure [35] using 4% (pH 6.8) stacking gel and 12% acrylamide separating gel (pH 8.8). A 2-fold dilution of the samples was dissolved in 2× Laemli sample buffer, containing 65.8 mM Tris-HCl (pH 6.8), 26.3% (w/v) glycerol, 2.1% SDS, 0.01% bromophenol blue and 2.5% (v/v) 2-mercaptoethanol. The solutions were boiled for 5 min before loading (20μL) onto the gel. After the electrophoresis, the gel sheet was fixed in 40% methanol, 10% acetic acid for 30 min. Then, the gel was washed in deionized water and stained by Bio-Safe Coomassie G-250 Stain solution (Bio-Rad, Hercules, USA).

### 2.5 Determination of antioxidant activities of BDLP extracts

#### 2.5.1 DPPH free radical scavenging activity

Method of Xie *et al.* [36] with slight modifications was followed. A sample of BDLP extract (1 mL) was added to 2 mL of 0.2 mM 2,2-diphenyl-1-picrylhydrazyl (DPPH) in 80% methanol. Then, vigorous mixing and keeping at room temperature in a dark place for 30 min A_517_ nm was measured by UV-vis spectrophotometer (UV1800; Shimadzu, Japan). A blank was prepared in the same manner, except that distilled water was used instead of the sample. Gallic acid was used to construct a standard curve. The activity was expressed as mg gallic acid equivalent (GAE) per 100 g dry basis of BDLP extract.

#### 2.5.2 ABTS radical scavenging activity

A method using a stable of free radical cation [ABTS•+; 2,2-azinobis-(3-ethylbenzo-thiazoline-6-sulphate] was modified from Re *et al.* [37] and Choi *et al.* [38]. An oxidant solution was prepared by mixing 2.45 mM of K_2_S_2_O_8_ and 7 mM ABTS solution dissolved in 20 mM sodium acetate buffer (pH 4.5), and then it was incubated at room temperature in the dark for 12–16 h to obtain a stable and dark blue-green radical solution. The oxidant solution was then diluted with 95% ethanol to an absorbance of 0.70 ± 0.02 at 734 nm and used as a working solution. Then, 20 μL of sample solution was added to 2 mL of the working solution, and the absorbance was measured at 734 nm after incubating the solution at room temperature in the dark for 6 min. The ABTS radical-scavenging activity was calculated against a standard curve of Trolox (0.1–1.0 mg/mL). Results were expressed as g Trolox equivalent (TE) per 100 g of BDLP extract.

#### 2.5.3 Ferric reducing antioxidant power (FRAP)

This test was assayed according to Benzie & Strain [39]. The FRAP solution consisted of 2.5 mL of 10 mM TPTZ solution in 40 mM HCl, 2.5 mL of 20 mM FeCl_3_.6H_2_O solution, and 20 mL of 300 mM acetate buffer (pH 3.6). The mixed solution was incubated at 37 °C for 30 min. The BDLP extract sample (50 μL) was added to the FRAP solution (750 μL) and kept for 30 min in the dark. Change in solution color was measured at 593 nm. A standard curve was prepared using FeSO_4_.7H_2_O and expressed as g FeSO_4_ equivalent per 100 g of BDLP extract.

### 2.6 Statistical analysis

Statistical analyses were performed using SPSS version 17.0 (SPSS Inc., Chicago, IL, USA). Results were reported as mean ± standard deviation (SD). Data were analyzed by one-way analysis of variance (ANOVA). Tukey's multiple comparisons test at a value *p* ≤ 0.05 was used to assess significant differences between means of samples.

## 3 Results and discussion

### 3.1 Effect on color characteristics of dried longan pulp

**Figure 2:**
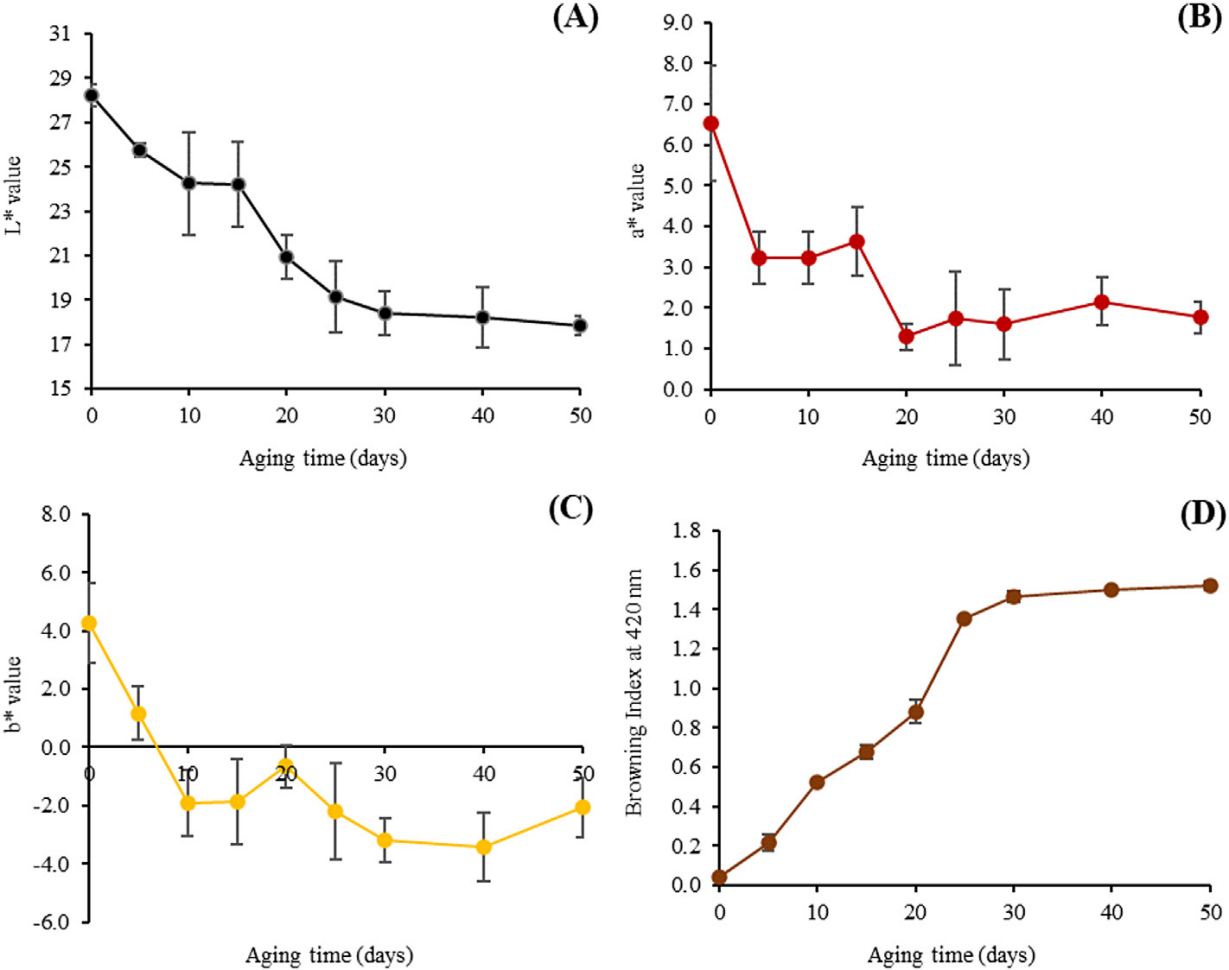
The change in average (A) lightness values (*L∗*); (B) redness values (*a∗*); (C) yellowness values (*b∗*) and (D) browning index (BI) of dried whole longan pulp during the aging period.

The color parameter of lightness (*L∗*) and the browning index (BI) of BDLP during 50 days aging time at 60 °C under a controlled relative humidity were shown in Figures 2A and 1D. The average of lightness values generally decreased as aging time increased, while the BI significantly increased during the first 30 days (*p* ≤ 0.05) and thereafter a constant absorbance during the remaining stages. The values of redness (*+a∗*) and yellowness (*+b∗*) showed a rapid decrease with the extension of the aging period for 5–10 days and afterwards slight alteration but the non-significant difference with increasing incubation time (*p* > 0.05) (Figures 2B and 2C). The BI level of dried longan pulp was increased due in part to *L∗*, *a∗*, and *b∗* value reduction.

Thus, the color of BDLP changed from light brown to black color. The color changes of longan pulp typically depended on the thermal process due to the non-enzymatic browning reaction, Maillard reaction, or the non-enzymatic glycosylation, which could correlate to a formation of brown pigments through an interaction between polysaccharides and protein molecules in longan pulp [13, 39, 40]. The factors affecting color development of non-enzymatic browning reaction were temperature, pH (between 6-10), water activity (0.3–0.7), and the concentration ratios of amino acid/sugar. Furthermore, the excess of reducing sugar over amino acid content could promote the browning value in the food system [41].

### 3.2 Effect on reducing sugar content and common/rare sugar composition

**Figure 3:**
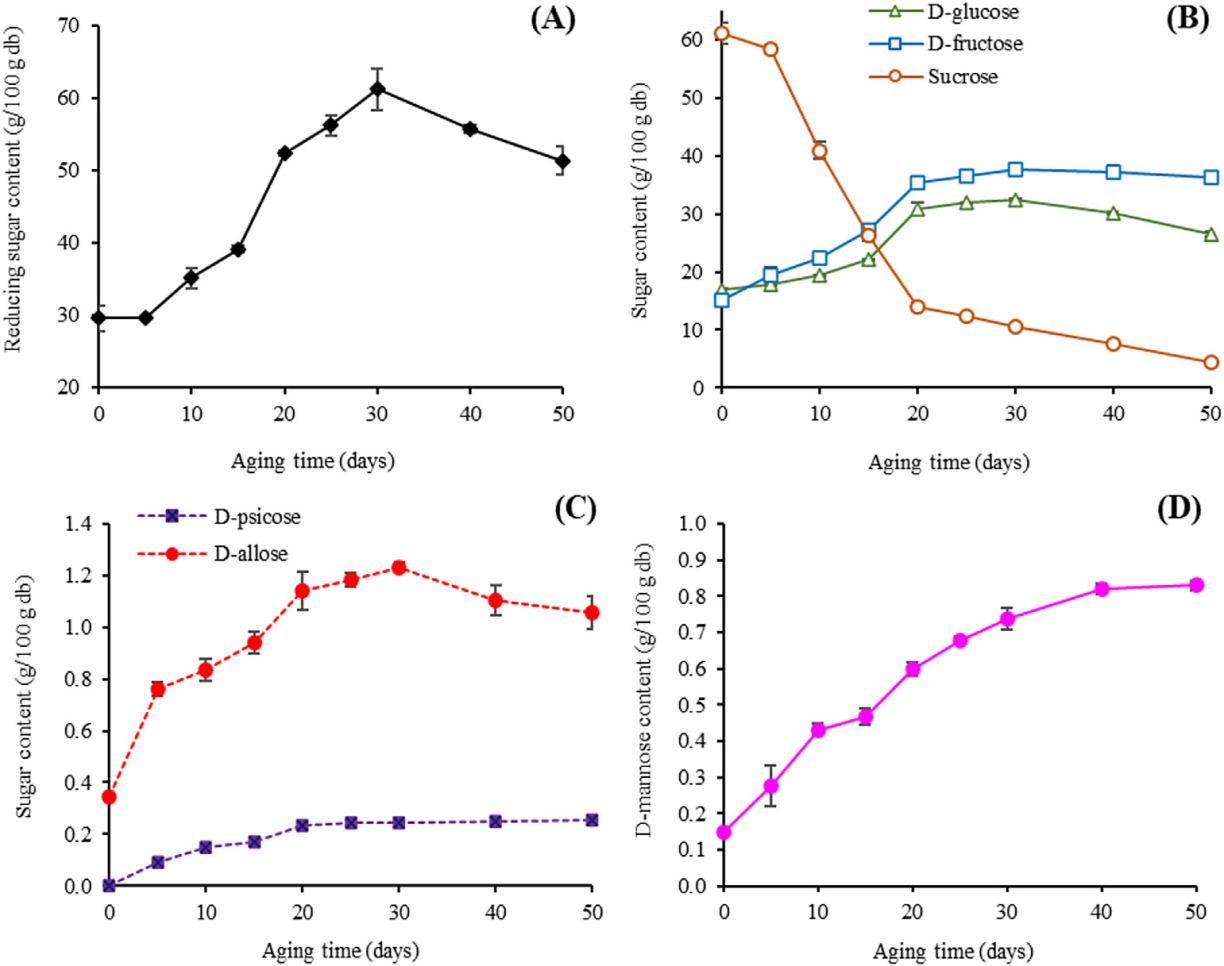
The change in average (A) reducing sugar content and (B) sugars content of D-glucose, D-fructose and sucrose (C) Rare sugar content (D-psicose and D-allose) and (D) D-mannose content of dried whole longan pulp during the aging period.

Figure 3A displays the reducing sugar content in BDLP during aging. The amount of reducing sugar gradually increased with incubation time. The average reducing sugar content of the control sample was 29.55 g/100 g dry basis, whereas that of 30 days of aging was 61.23 g/100 g dry basis, and thereafter significantly decreased during the remaining period (*p* ≤ 0.05). The level of common mono/disaccharides sugars (D-glucose, D-fructose, and sucrose) in dried whole longan pulp during incubation time was showed in Figure 2B. Focusing on the change of sucrose was decreased 15-fold after 50 days of aging. By contrast, the D-glucose and D-fructose contents moderately increased in which the D-fructose concentration was 1.3-fold higher than that of D-glucose. However, the content of these sugars in BDLP after 30 days decreased during the period considered. The values of D-glucose and D-fructose ranged from 17.04-32.39 g/100 g dry basis and 15.02–37.71 g/100 g dry basis, respectively.

Generally, the most abundant sugars in longan pulp were sucrose, D-fructose, and D-glucose [42]. However, this study found the minimal value of sucrose in BDLP after aging for 20 days. It may be caused by thermal degradation of sucrose molecule which could split the glycosidic bond, resulting in the increasing of D-glucose and D-fructose formation (Figure 3B). This reaction was known as Lobry de Bruyn-Alberda van Ekenstein rearrangement or LA-transformation [25, 43, 44, 45]. Owing to Maillard reaction, the order of sugar reactivity has been reported that glucose was more reactive than fructose towards amino acid by non-covalent hydrophobic interactions or/and by covalent disulfide bonds [46, 47, 48]. This might explain why the amount of D-glucose in BDLP was less than D-fructose after the aging.

The rare sugar types (e.g., D-psicose and D-allose) were investigated in all BDLP samples (Figure 3C). It was found that D-psicose developed during 20 days of aging time (*p* ≤ 0.05) and remained rather constant for the remained time. The concentration of D-allose was also identified and found greatly increasing during the aging period of 30 days and thereafter it slightly decreased (1.23–1.06 g/100 g dry basis). Moreover, there was an increase in D-mannose concentration during the incubation time (ranging from 0.15-0.83 g/100 g dry basis) as shown in Figure 3D. Therefore, it was established that D-psicose, D-allose and D-mannose increased over 2, 3, and 5-fold in BDLP samples during 50 days of aging compared to those in the control sample (aging time = 0) in which the sugar contents were approximately 0.10, 0.34, and 0.15 g/100 g dry basis, respectively. However, D-xylose and D-rhamnose did not show in any sample.

Some rare sugars, i.e., D-psicose and D-allose had been reported previously in dried longan pulp samples. Oshima *et al.* [26] suggested that these types of sugar could also be found in various food products containing high fructose content. A study on D-psicose and D-allose formation indicated that it might be caused by glycosylation/non-enzymatic browning reaction during cooking at high temperatures, high pH, high fructose concentration, and/or by a prolonged cooking time. In addition, the thermal processing could also influence some rare sugars (D-psicose, D-allose) obtaining by enolization/isomerization of LA-transformation of glucose and fructose structure configuration. The LA-transformation was the base or acid catalyzed transformation of an aldose to ketose isomer, which the 1,2-enediol anion was an intermediate stage of monosaccharides isomerization reaction. Additionally, the 1,2-Enolization reaction of glucose molecule may transform to fructose and/or mannose molecule as well [25, 49, 50].

### 3.3 Effect on amino acid content and degree of glycation

The data on amino acids in BDLP extract separated by HPLC was shown in Table 1. The major amino acid in dried longan pulp (control sample) was glutamic acid (93.83 μg/g dry basis), followed by alanine and cystine (33.48 μg/g dry basis), and leucine (28.52 μg/g dry basis). Other amino acids with low quantity were histidine, methionine, serine, arginine, phenylalanine, proline, isoleucine, glycine, valine, threonine, tyrosine, and lysine. Their values were in the range of 0.49–7.99 μg/g dry basis. Only aspartic acid content was not detectable in all of the dried longan samples. The effect of aging time on amino acid composition was evaluated. It was observed that there was a significant reduction in each amino acid content during the aging period (*p* ≤ 0.05). Threonine was completely lost after 15 days of incubating. Three amino acids were not detected after 20 days of aging that were threonine, tyrosine, and lysine. Moreover, glutamic acid and arginine significantly decreased with the aging time which their contents were unable to recognize on the 30 days of the aging period. During the remaining period, the amount of isoleucine, proline, glycine, valine, methionine, phenylalanine, and histidine were not found in BDLP samples. Nevertheless, the compositions of serine, alanine + cystine, and leucine were still observable in the pulp although the dried pulp samples were aged for 50 days. According to Table 1, the percentage of remaining amino acids was remarkably decreased with increasing incubating time due to Maillard reaction or glycosylation between terminal α-or ϵ-amino functions brought by amino acids of peptides and the carbonyl function of reducing sugars to produce the SPC [48, 51, 52]. Besides, the reduction of amino acid might be caused by an interaction between phenolic compounds and protein via non-covalent physical interactions such as electrostatic, hydrophobic, van der Waals, and hydrogen bonding [53, 54].

**Table 1:** Composition (μg/g dry basis) of free amino acids in dried whole longan pulp during the aging period.

Amino acids (μg/g dry basis)	Aging period (days)								
	0	5	10	15	20	25	30	40	50
Aspartic acid	ND	ND	ND	ND	ND	ND	ND	ND	ND
Threonine	1.46 ± 0.06^a^	1.38 ± 0.01^b^	1.37 ± 0.02^b^	ND	ND	ND	ND	ND	ND
Serine	5.59 ± 0.21^a^	4.52 ± 0.03^b^	4.34 ± 0.23^b^	4.72 ± 0.07^b^	4.33 ± 0.53^b^	3.84 ± 0.04^bc^	3.32 ± 1.36^c^	1.10 ± 0.01^d^	0.30 ± 0.10^d^
Glutamic acid	93.83 ± 4.23^a^	29.29 ± 3.68^b^	12.47 ± 0.24^c^	7.38 ± 0.53^d^	4.35 ± 0.16^de^	3.53 ± 1.35^e^	ND	ND	ND
Proline	4.63 ± 0.01^a^	3.69 ± 0.19^b^	3.02 ± 0.04^c^	3.05 ± 0.05^c^	2.71 ± 0.30^d^	2.10 ± 0.11^e^	1.36 ± 0.15^f^	0.69 ± 0.01^g^	ND
Glycine	2.33 ± 0.02^a^	1.96 ± 0.11^b^	1.39 ± 0.01^c^	1.07 ± 0.10^d^	0.91 ± 0.03^e^	0.75 ± 0.15^f^	0.49 ± 0.08^g^	0.34 ± 0.01^h^	ND
Alanine+Cystine	33.84 ± 1.55^a^	30.08 ± 2.89^b^	14.94 ± 1.07^c^	11.74 ± 1.06^d^	7.65 ± 0.32^e^	5.28 ± 0.15^f^	3.85 ± 0.19^fg^	2.66 ± 0.04^gh^	1.27 ± 0.25^h^
Valine	1.99 ± 0.15^a^	1.55 ± 0.46^b^	1.79 ± 0.02^ab^	1.53 ± 0.15^b^	1.10 ± 0.03^cd^	1.17 ± 0.23^c^	0.79 ± 0.07^de^	0.72 ± 0.04^e^	ND
Methionine	6.49 ± 0.10^a^	5.33 ± 0.71^b^	1.63 ± 0.44^c^	1.47 ± 0.04^c^	0.94 ± 0.01^d^	0.82 ± 0.08^de^	0.35 ± 0.22^ef^	0.14 ± 0.01^f^	ND
Isoleucine	4.25 ± 0.01^a^	2.58 ± 0.65^b^	1.82 ± 1.01^bc^	2.00 ± 1.20^bc^	1.32 ± 0.03^c^	0.97 ± 0.11^cd^	0.19 ± 0.06^d^	ND	ND
Leucine	28.52 ± 0.02^a^	23.67 ± 5.19^b^	20.10 ± 1.78^c^	17.30 ± 0.22^cd^	14.20 ± 0.65^d^	8.85 ± 0.23^e^	9.97 ± 0.31^e^	3.86 ± 0.07^f^	1.33 ± 0.15^f^
Tyrosine	1.29 ± 0.12^a^	1.15 ± 0.64^a^	0.87 ± 0.88^a^	0.60 ± 0.02^b^	ND	ND	ND	ND	ND
Phenylalanine	4.87 ± 0.72^a^	5.62 ± 0.35^a^	4.01 ± 0.09^ab^	2.42 ± 0.33^bc^	1.66 ± 0.05^cd^	1.40 ± 0.08^cd^	0.89 ± 0.03^cd^	0.76 ± 0.14^cd^	ND
Histidine	7.99 ± 0.31^a^	6.83 ± 0.14^b^	4.79 ± 0.38^c^	3.72 ± 0.10^d^	3.90 ± 0.12^d^	2.83 ± 0.02^e^	2.09 ± 0.06^f^	1.17 ± 0.14^g^	ND
Lysine	0.49 ± 0.04^a^	0.09 ± 0.03^b^	0.03 ± 0.01^c^	0.02 ± 0.01^cd^	ND	ND	ND	ND	ND
Arginine	5.27 ± 0.27^a^	1.69 ± 0.18^b^	0.89 ± 0.35^c^	0.51 ± 0.25^d^	0.04 ± 0.01^e^	0.03 ± 0.01^e^	ND	ND	ND
Total amino acids	202.84	119.43	73.46	57.53	43.11	31.57	23.30	11.44	2.90
Remaining amino acids (%)	100	58.88	36.22	28.36	21.25	15.56	11.49	5.64	1.43

Note: Data represented as means ± SD (n = 3); ^a-h^ Mean values within each row with different superscript letters were significantly different (*p* ≤ 0.05); ND: not detected; Total amino acids indicated sum of 17 amino acids; Remaining amino acids (%) = (Total amino acids at *t* days × 100)/Total amino acids at 0 day.

**Figure 4:**
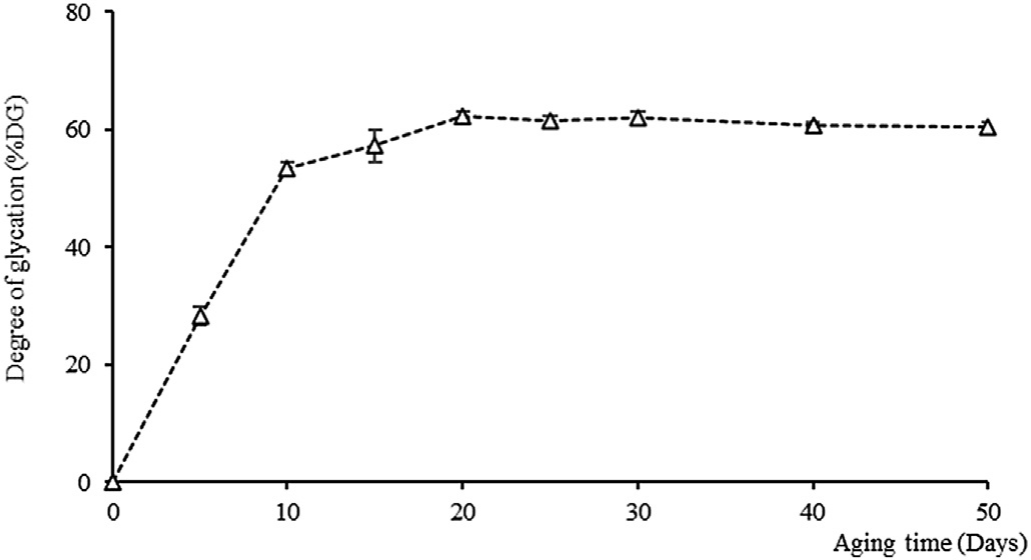
The change in average degree of glycation of dried whole longan pulp during the aging period.

The untreated dried whole longan was used as the control (A_0_) and was defined as no number of conjugations. The degree of glycation (DG) was determined by OPA measurements based on the reaction between the free amino group of the protein amino acid side-chain and the carbonyl group at the end of polysaccharide or/and reducing sugar molecules. The reduction of free amino acid group can indicate an increase of glycosylation grafting reaction which can be quantified by the DG [33]. The DG of BDLP conjugates obtained under different treatment conditions was shown in Figure 4. The DG increased with the aging time until 20 days and afterwards the level of DG remained constant around 60–62% (*p* > 0.05). The graft reactions between amino acids and reducing sugars in longan pulp were conducted by the moist-dry-heating process. These results correlated with the reduction of amino acid and monosaccharide contents on previous analysis. Several investigations have shown that the SPC exhibited DG extension with the increasing of BI and also acidity during the heating time. In many researches, SPC can provide beneficial effects on the biochemical activities and functional properties [12, 15, 55].

### 3.4 Evaluation of conjugate formation by SDS-PAGE

**Figure 5:**
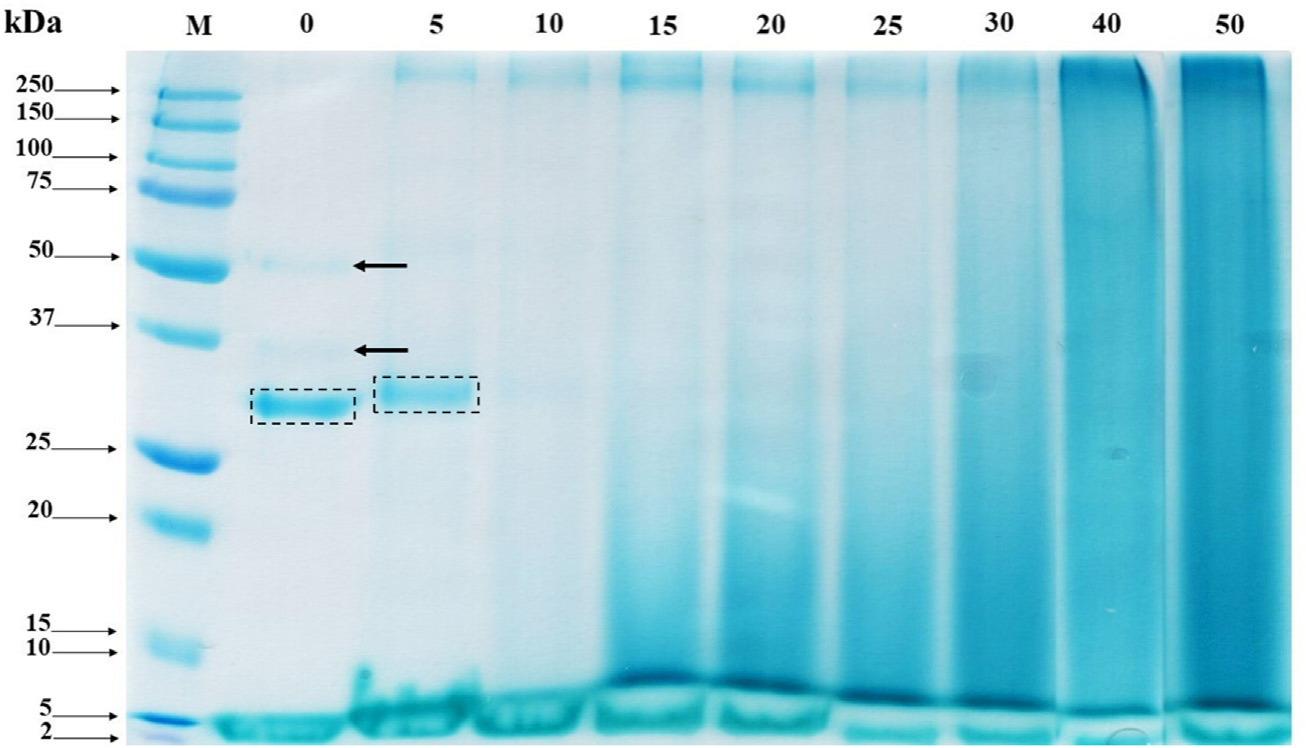
SDS-PAGE patterns of BDLP obtained at 60 °C under controlled relative humidity (75%) during the aging period (Lane M: protein standards; Lane 0–50: aging time, day).

To establish the covalent binding of SPC and other conjugated compounds, the SDS-PAGE profiles of BDLP extracts was performed (Figure 5). The molecular weight (MW) of protein molecules in longan pulp (control sample) was obtained one intense band at ~31 kDa and another two pallid bands at ~36 kDa and ~50 kDa. The SDS-PAGE pattern showed an increase in the amount of high molecular weight (HMW) pattern over the standard band of 250 kDa during the aging time of dried whole longan. In addition, the decrease in band intensity could indicate a formation of HMW molecules or conjugated compounds that were too large to enter the gel. Thus, a large majority of the protein bands were found on the top of the gel. The glycated protein smear band was slightly developed on the 5 days of aging. Moreover, this smear band was shifted to a higher molecular mass and was faded as the aging time increased. The conjugates molecules migrated slower and broader in an SDS-PAGE gel which could indicate the glycation [56, 57, 58] Similar to studies by Oh *et al.* [59] and Li, Arunkumar, & Etzel [60], they observed that the band intensity of major proteins was decreased during glycosylation, leading to a new smearing pattern of conjugated compounds. However, when comparing the DG with the high glycosylated molecules formation during aging period, it was found that the constant value of DG by OPA method during the remaining stages correlated to HMW band appearance on SDS-PAGE gel. The OPA method was determined only available amino group per molecule and did not able to distinguish between glycosylated and non-glycosylated molecules [61, 62]. Nevertheless, the gradual disappearance of dried longan control bands indicated a formation of aggregates upon the heating time due to the occurrence of non-enzymatic glycosylation or Maillard reaction. Besides, the smearing zone was also obtained in all BDLP extracts. The impurification of the extracts might have some effect on the protein-stained electrophoretic pattern, resulting in the appearance of a smearing zone of MRPs at HMW on a gel [63]. Moreover, due in part to their increased molecular size of an Amadori product from Maillard reaction, this could cause difficulty in ionizing proteins as well as protein aggregation while running gel [64]. The wide-spread of molecular weight might also attribute to the heterogeneous distribution of SPC components which were a diverse array of protein molecular weight after the moist-dry-heating process. However, this research was tested in the complex compositions of dried longan pulp without protein purification, that the intense bands might be the Maillard conjugates and/or the other macromolecules, which were found naturally in fruit.

### 3.5 Effect on antioxidant activity

**Figure 6:**
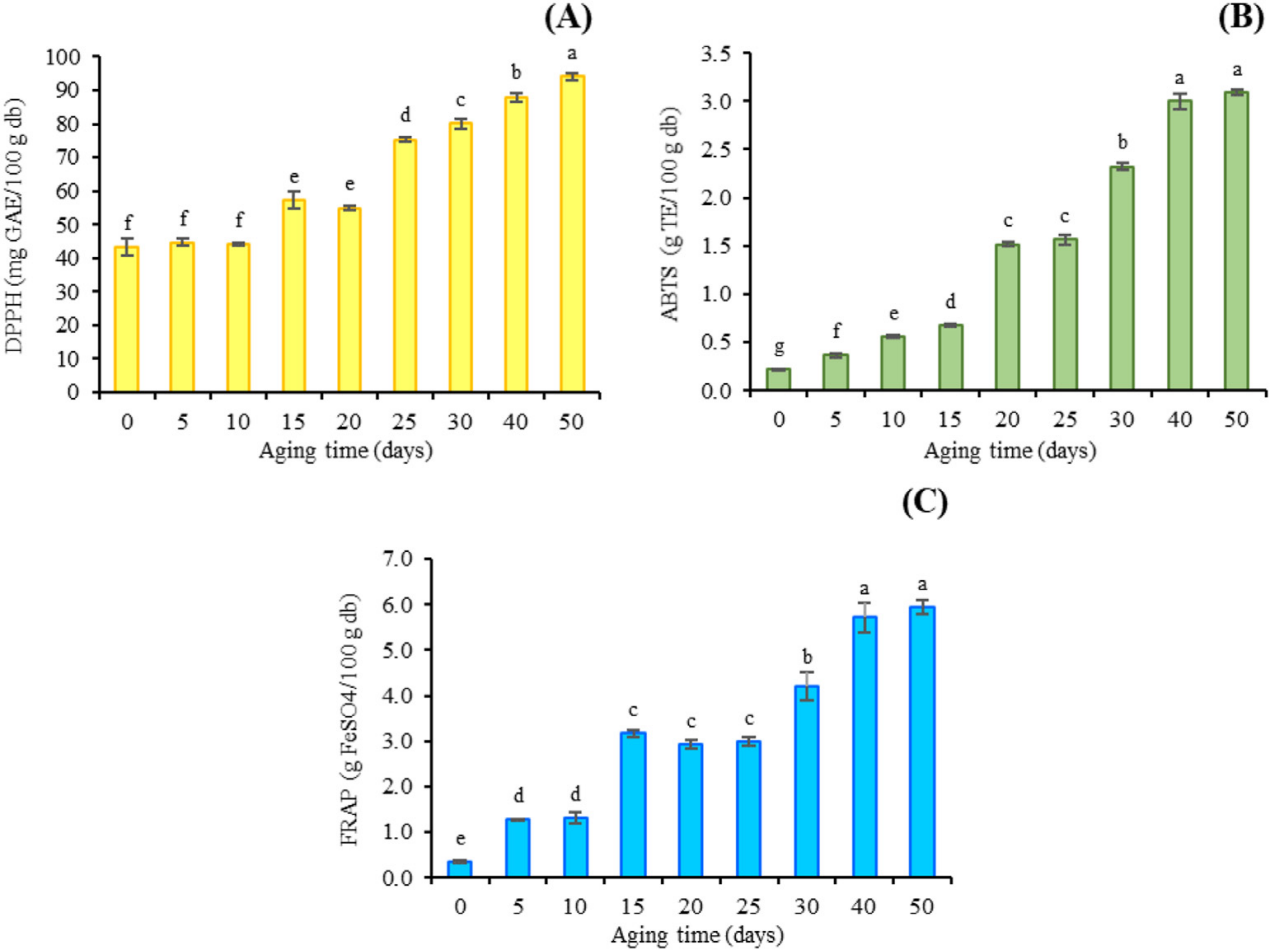
The change in the average of antioxidant activities: (A) DPPH free radical scavenging activity, (B) ABTS radical scavenging activity, and (C) FRAP reducing antioxidant power of dried whole longan pulp during the aging time.

The methods of antioxidant capacity measurements were achieved. DPPH and ABTS radical scavenging photometric assays were described by the corresponding substrates to undergo single electron and hydrogen atom transfer ability by two components in the reaction mixture (Figures 6A and 6B). The DPPH and ABTS•+ radical scavenging of BDLP after 20 days of aging were significantly higher than that of a control sample which the activities increased approximately 1.3-fold (54.94 mg GAE/100 g dry basis) and 6.9-fold (1.52 g TE/100 g dry basis), respectively. The antioxidant activity of BDLP under the moist-dry-heat process gradually increased with the incubation period. The FRAP assay, a method on basis of the reduction of ferric (Fe^3+^) to ferrous (Fe^2+^) was also determined. As shown in Figure 6C, the FRAP values of BDLP also increased similar to those of DPPH and ABTS. The FRAP value of BDLP on 20 days of aging time increased approximately 9-fold (2.92 g FeSO_4_/100 g dry basis) when compared to the untreated dried longan pulp. Moreover, the results of all antioxidant assays displayed a positive correlation with the aging time. The activities were in the range of 43.20–94.11 mg GAE/100 g dry basis for DPPH, 0.23–3.09 g TE/100 g dry basis for ABTS, and 0.35–5.95 g FeSO_4_/100 g dry basis for FRAP. Several studies suggested that due to their high thermostability, MRPs/SPC/other conjugate compounds could be used in processed-thermal products with potential antioxidant property. The antioxidant activity of SPC has been involved their pyrrole and hydroxyl groups, generating radical chain reactions. Besides, the conjugated compounds exhibited their antioxidant effect through metal chelate compounds, hydrogen peroxide degrading effect, and reactive oxygen entrapping property [16, 65, 66]. The increased reducing power of conjugates may attribute to the electron transferring, thermal denatured protein and amino acid that could be able to transfer electrons [16, 67]. Additionally, the rare sugar effect especially D-psicose and D-allose could provide an effective role in Maillard reaction to increase the antioxidant capacity [17, 68, 69].

## 4 Conclusion

Black dried longan pulp (BDLP), produced by aging dried whole longan at 60 °C in 75% relative humidity for 50 days, showed higher antioxidant properties, a lower sucrose level, and more rare sugar formation than the untreated dried longan pulp. Moreover, the maximum BI value and a minimum lightness value of BDLP were found on the 30 days of aging time in which the pulp color changed from light brown to black color. The amount of reducing sugar gradually increased with the incubation period whereas it slightly decreased after 30 days of incubation. The rare sugars, D-psicose and D-allose was present in all BDLP and D-mannose also gradually increased during reaction time. Different incubation times of the moist-dry-heat process affected the reduction of free amino acid content and the grafting ability between amino acid groups and sugar groups. The HMW band appearance on SDS-PAGE gel confirmed the presence of conjugated compounds. The antioxidant qualities of BDLP displayed a positive correlation with the aging period. Thus, the dry-heat treatment with high relative humidity control was proven to be an effective process for improving the antioxidant activity of black dried longan pulp product.

## Declarations

### Author contribution statement

Chalermkwan Somjai: Conceived and designed the experiments; Performed the experiments; Analyzed and interpreted the data; Wrote the paper.

Thanyaporn Siriwoharn, Kanokwan Kulprachakarn: Conceived and designed the experiments; Wrote the paper.

Supakit Chaipoot, Rewat Phongphisutthinant: Contributed reagents, materials, analysis tools or data.

Pairote Wiriyacharee: Conceived and designed the experiments; Analyzed and interpreted the data; Contributed reagents, materials, analysis tools or data; Wrote the paper.

### Funding statement

This work was supported by 10.13039/501100004704National Research Council of Thailand (NRCT) Research Grant, 2020.

### Data availability statement

Data included in article/supplementary material/referenced in article.

### Declaration of interests statement

The authors declare no conflict of interest.

### Additional information

No additional information is available for this paper.
